# Lobaplatin-based concurrent chemoradiotherapy in elderly nasopharyngeal carcinoma

**DOI:** 10.1080/07853890.2024.2383959

**Published:** 2024-07-31

**Authors:** Yi-Feng Yu, Ping Zhou, Rui Zhou, Qin Lin, San-Gang Wu

**Affiliations:** Department of Radiation Oncology, Xiamen Cancer Quality Control Center, Xiamen Cancer Center, Xiamen Key Laboratory of Radiation Oncology, the First Affiliated Hospital of Xiamen University, School of Medicine, Xiamen University, Xiamen, China

**Keywords:** Nasopharyngeal carcinoma, radiotherapy, lobaplatin, treatment response, survival, radiotherapy

## Abstract

**Background:**

The therapeutic benefit of concurrent chemoradiotherapy (CCRT) in elderly nasopharyngeal carcinoma (NPC) patients remains controversial. This study aimed to investigate the efficacy and toxicity of lobaplatin-based CCRT in elderly patients with NPC.

**Methods:**

We included stage II–IVA NPC patients aged ≥65 years who received lobaplatin concomitant with intensity-modulated radiation therapy (IMRT) between March 2019 and January 2023. Objective response rates and treatment-related toxicity were assessed. Kaplan–Meier’s analysis was performed to calculate survival rates.

**Results:**

A total of 29 patients were included with a median age of 67 years. There were 19 patients (65.5%) who had comorbidities. All patients had serum EBV-DNA detective before treatment; the median EBV-DNA load was 236 IU/mL. There were 25 (86.2%) patients treated with induction chemotherapy, and the overall response rate was 92.0%. All patients received IMRT and concurrent chemotherapy with lobaplatin. During the CCRT, the most common adverse effect was haematological toxicity. Three patients (10.3%) had grade 3 leucopenia, three patients (10.3%) had grade 3 neutropenia, and eight patients (27.6%) had grade 3–4 thrombocytopenia. The rate of grade 3 mucositis was 34.5%. No patients had liver and kidney dysfunction. The median weight loss was 4 kg during CCRT. After three months of CCRT, the total response rate was 100%. EBV-DNA was not detected in any patients. The median follow-up was 32.1 months. The 3-year locoregional recurrence-free survival, distant metastasis-free survival, progression-free survival and overall survival were 95.8%, 85.7%, 82.5% and 100%, respectively.

**Conclusions:**

Lobaplatin-based CCRT is safe and feasible for elderly NPC patients, with satisfactory short-term survival outcomes and acceptable toxicities. A phase 2 trial is ongoing to investigate the role of lobaplatin-based CCRT on long-term survival and treatment toxicities for this population.

## Introduction

Nasopharyngeal carcinoma (NPC) is an epithelial malignancy characterized by unique geographical distribution, with the highest prevalence in southeast China and Southeast Asia [1]. It is also important to note that the age distribution of NPC is also unique. In China, the age-specific incidence of NPC increases with age peaks at 60–64 years, and begins to decline thereafter [2]. As life expectancy increases, there may be more patients diagnosed with NPC in the elderly [3]. Compared to younger patients, elderly patients have a higher risk of anticancer treatment toxicity due to comorbidities, as well as suboptimal organ function, nutritional status and/or social support [4–6]. Several studies including ours have found that elderly NPC patients had inferior survival outcomes compared to younger NPC patients [7,8]. However, treatment guidelines for elderly NPC patients are lacking. In addition, elderly patients with NPC were either excluded or accounted for only a few patients in most clinical trials [9–12]. Therefore, the optimal management for elderly NPC patients remains unclear.

Due to the insidious nature of NPC, approximately 70–80% of NPC patients are diagnosed with locally advanced diseases (stage III–IVA disease) [13]. Several studies have shown that the pattern of stage distribution at NPC diagnosis in elderly patients was similar to the general population [14,15]. In the current National Comprehensive Cancer Network and Chinese Society of Clinical Oncology guidelines, the optimal treatment for locally advanced NPC is induction chemotherapy (IC) + cisplatin-based concurrent chemoradiotherapy (CCRT) [16,17]. However, the therapeutic benefit of CCRT in elderly NPC patients remains controversial. Several retrospective studies have shown that the addition of cisplatin to intensity-modulated radiation therapy (IMRT) was not associated with better survival outcomes but with higher toxicities compared to those treated with IMRT alone [18–20]. Death due to treatment-related toxicity is more likely in elderly patients, which may compensate for the benefit of CCRT in this population [15,21]. Several prospective phase 3 studies have confirmed that the alternative platinum derivatives nedaplatin- or lobaplatin-based CCRT have non-inferior progression-free survival (PFS) and lower treatment-related toxicity compared to cisplatin-based CCRT [22,23]. A previous phase 3 trial demonstrated comparable 5-year rates of locoregional relapse-free survival (LRFS) (87.7% vs. 88.8%), distant metastasis-free survival (DMFS) (86.6% vs. 85%), PFS (74.8% vs. 76.4%) and overall survival (OS) (88.2% vs. 89%) between those treated with lobaplatin-based and cisplatin-based CCRT. In addition, the lobaplatin-based regimen exhibited a lower incidence of gastrointestinal toxicity, nephrotoxicity and weight loss compared to the cisplatin-based CCRT group [23]. However, it is worth noting that the abovementioned study only enrolled patients under 60 years old, so the impact of lobaplatin-based CCRT on the elderly population remains uncertain. To our knowledge, there are no studies on the efficacy and toxicity of platinum derivatives in the elderly with NPC. In this study, we aimed to investigate the efficacy and toxicity of lobaplatin-based CCRT in elderly patients with NPC.

## Materials and methods

### Patients

A prospective study published in 2018 showed that lobaplatin-based CCRT had a positive effect and was well tolerant in locally advanced NPC [24]. We started using lobaplatin in elderly NPC patients in 2019. This study included NPC patients aged ≥65 years receiving lobaplatin-based CCRT between March 2019 and January 2023 in our institution. Patients who met the following criteria were included: (1) age ≥65 years; (2) stage II–IVA NPC patients; (3) received IMRT with concurrent lobaplatin-based chemotherapy; (4) complete clinicopathologic and treatment data. A total of 29 patients were included in our study. This study was approved by the Institutional Review Boards of the First Affiliated Hospital of Xiamen University (No. KYH2019-045) and written informed consent was obtained from the study participants before study commencement.

### Pre-treatment assessment

All patients undergo baseline assessment before initiating treatment, including a complete history and physical examination, nasopharyngoscope, baseline blood tests and serum Epstein-Barr virus (EBV)-DNA load. Three patients (23.1%) underwent nasopharyngeal and neck magnetic resonance imaging (MRI), chest computer tomography (CT) scan, abdominal ultrasound as well as a bone scan. Ten patients (76.9%) underwent nasopharyngeal and neck MRI and PET/CT scans. All patients were staged according to the eighth edition of the American Joint Committee on Cancer staging system. The severity of comorbidity at diagnosis was determined by the age-adjusted Charlson comorbidity index (ACCI) [25].

### Radiotherapy

The gross target volume for primary nasopharyngeal tumour (GTVnx) and neck positive lymph nodes (GTVnd) included all known gross diseases determined by clinical examination and imaging findings. The delineation of the clinical target volume (CTV) was based on the recommendation from the consensus guidelines by the international experts [26]. In this study, all patients receive radical IMRT with a simultaneous integrated boost to the primary nasopharyngeal tumour and involved neck lymph nodes. The plan target volume (PTV) was constructed automatically with an additional 3-mm margin in three dimensions for each volume to account for set-up variability. The dose to PGTVnx and PGTVnd was treated to a dose of 70.29 Gy in 33 fractions with 2.13 Gy/fractions, PCTV1 and PCTVnd (high-risk region) were treated to a dose of 62.14 Gy in 33 fractions with 1.88 Gy/fractions, and PCTV2 (low-risk region) was treated to a dose of 56.10 Gy in 33 fractions with 1.70 Gy/fractions. All treatment was delivered once daily, five days per week.

### Chemotherapy

Whether the patient receives IC was determined by the clinician. The IC regimen was not protocolized and was used at the discretion of the clinician of individual cases. A total of 25 patients (86.2%) were treated with IC. The concurrent chemotherapy regimen consisted of three-weekly lobaplatin administered for two cycles, starting on the initial date of IMRT. Lobaplatin was administered at 30–40 mg/m^2^ based on the previous dose–escalation study [24].

### Treatment responses assessment and follow-up

The assessment of tumour response was performed after the completion of IC, one and three months after CCRT, which was based on MRI and nasopharyngoscope according to Response Evaluation Criteria for Solid Tumours criteria. Adverse effects regarding IC were graded by the National Cancer Institute Common Toxicity Criteria (version 3.0), whereas CCRT-induced toxicities were scored based on the Acute and Late Radiation Morbidity Scoring Criteria of the Radiation Therapy Oncology Group. Patients were followed up every three months. Each follow-up included a careful examination of the blood tests, EBV-DNA load, the nasopharynx and neck nodes by an experienced doctor, an MRI scan of the nasopharynx and neck lymph node, nasopharyngoscope, chest CT scan and abdominal ultrasound. LRFS was measured as the duration time from the diagnosis of NPC to the occurrence of relapse in the nasopharynx or neck lymph nodes or until the last follow-up. DMFS was defined as the duration time of NPC diagnosis to the time of distant metastasis or the last follow-up. PFS was referred to as the time from NPC diagnosis to the date of disease progression. OS was defined as the time of NPC diagnosis until death from any cause.

### Statistical analysis

SPSS statistical software (version 25.0, IBM Corporation, Armonk, NY) was used to perform the data analysis, and treatment response and adverse events were presented as descriptive statistics. Survival curves were calculated using Kaplan–Meier’s analysis.

## Results

### Patient characteristics

A total of 29 patients were included with a median age of 67 years (range, 65–75 years). The characteristics of patients are shown in Table 1. Of these patients, 69.0% (*n* = 20) were male and 86.2% (*n* = 25) were stage III–IVA disease. Regarding histology, 27 (93.1%) patients had the WHO III subtype and two (6.9%) patients had the WHO II subtype. There were 19 patients (65.5%) who had ACCI ≥3. The most common comorbidities were diabetes and hypertension, followed by a previous malignant tumour. All patients had serum EBV-DNA detective before treatment, the median EBV-DNA load was 236 IU/mL (range, 73–6.97 × 10^5^ IU/mL).

**Table 1.** Patient baseline characteristics.

Variables	*n* (%)
Gender	
Male	20 (69.0)
Female	9 (31.0)
ACCI	
<3	10 (34.5)
≥3	19 (65.5)
Histology	
WHO II	2 (6.9)
WHO III	27 (93.1)
Tumour stage	
T1	1 (3.4)
T2	4 (13.8)
T3	17 (58.6)
T4	7 (24.1)
Nodal stage	
N0	2 (6.9)
N1	16 (55.2)
N2	8 (27.6)
N3	3 (10.3)
Clinical stage	
II	4 (13.8)
III	15 (51.7)
IVA	10 (34.5)
Induction chemotherapy	
No	4 (13.8)
Yes	25 (86.2)

### Treatment

There were 25 patients (86.2%) treated with IC, the most commonly used chemotherapy regimens were gemcitabine + cisplatin (*n* = 8), followed by albumin-bound paclitaxel + nedaplatin (*n* = 5), albumin-bound paclitaxel + cisplatin (*n* = 4), gemcitabine + nedaplatin (*n* = 2), gemcitabine + S1 (*n* = 3) and docetaxel + lobaplatin (*n* = 1). The median IC cycles were 3 (range, 2–3 cycles). There were 22 patients (88.0%) who received pegylated granulocyte colony-stimulating factor to prevent myelosuppression.

All patients received IMRT and concurrent chemotherapy with lobaplatin. Of these patients, two cycles of concurrent lobaplatin chemotherapy were used.

### Treatment toxicity

During the CCRT, the most common adverse effect was haematological toxicity. Three patients (10.3%) had grade 3 leucopenia, three patients (10.3%) had grade 3 neutropenia, and eight patients (27.6%) had grade 3–4 thrombocytopenia. The grade 3 mucositis was 34.5%. Only two patients (6.9%) had nausea/vomiting during CCRT. Oral mucositis, anorexia and dermatitis were common non-haematological toxicities. No patients had liver and kidney dysfunction. The treatment toxicities by IC and CCRT are shown in Table 2. The median weight loss was 4 kg during CCRT (range, 0–7 kg). During the CCRT, 15 patients (51.7%) completed CCRT as required, and 14 patients (48.3%) had radiotherapy interrupted due to grade 3–4 haematological toxicity or mucositis. Among those with radiotherapy interrupted (*n* = 14), five patients (35.7%) had an interruption time of less than five days, eight patients (57.1%) had an interruption time of 5–10 days, and one patient (7.1%) had an interruption time of more than 10 days. There were 79.3% of patients who received the full dosage of lobaplatin.

**Table 2.** The treatment-related toxicities during concurrent chemoradiotherapy.

Toxicities	Grade 1–2	Grade 3	Grade 4
Leucopenia	15 (51.7%)	3 (10.3%)	0 (0.0%)
Neutropenia	10 (34.5%)	3 (10.3%)	0 (0.0%)
Thrombocytopenia	8 (27.6%)	6 (20.7%)	2 (6.9%)
Anaemia	21 (72.4%)	3 (10.3%)	0 (0.0%)
Anorexia	27 (93.1%)	0	0
Fatigue	26 (89.7%)	0	0
Dermatitis	25 (86.2%)	2 (6.9%)	0
Dry mouth	27 (93.1%)	2 (6.9%)	0
Oral mucositis	19 (65.5%)	10 (34.5%)	0
Liver dysfunction	0	0	0
Kidney dysfunction	0	0	0
Nausea/vomiting	2 (6.9%)	0	0

### Treatment responses and survival

The overall response rate to IC was 92.0% (*n* = 23). Regarding primary nasopharyngeal tumour, five patients (20.0%) had complete remission (CR) and 19 patients (76.0%) had partial remission (PR) after IC. There were also four patients (16.0%) who had CR and 20 patients (80.0%) who had PR in regional lymph nodes after IC. The load of EBV-DNA was decreased to 0 in all patients who completed the IC.

**Figure 1.**
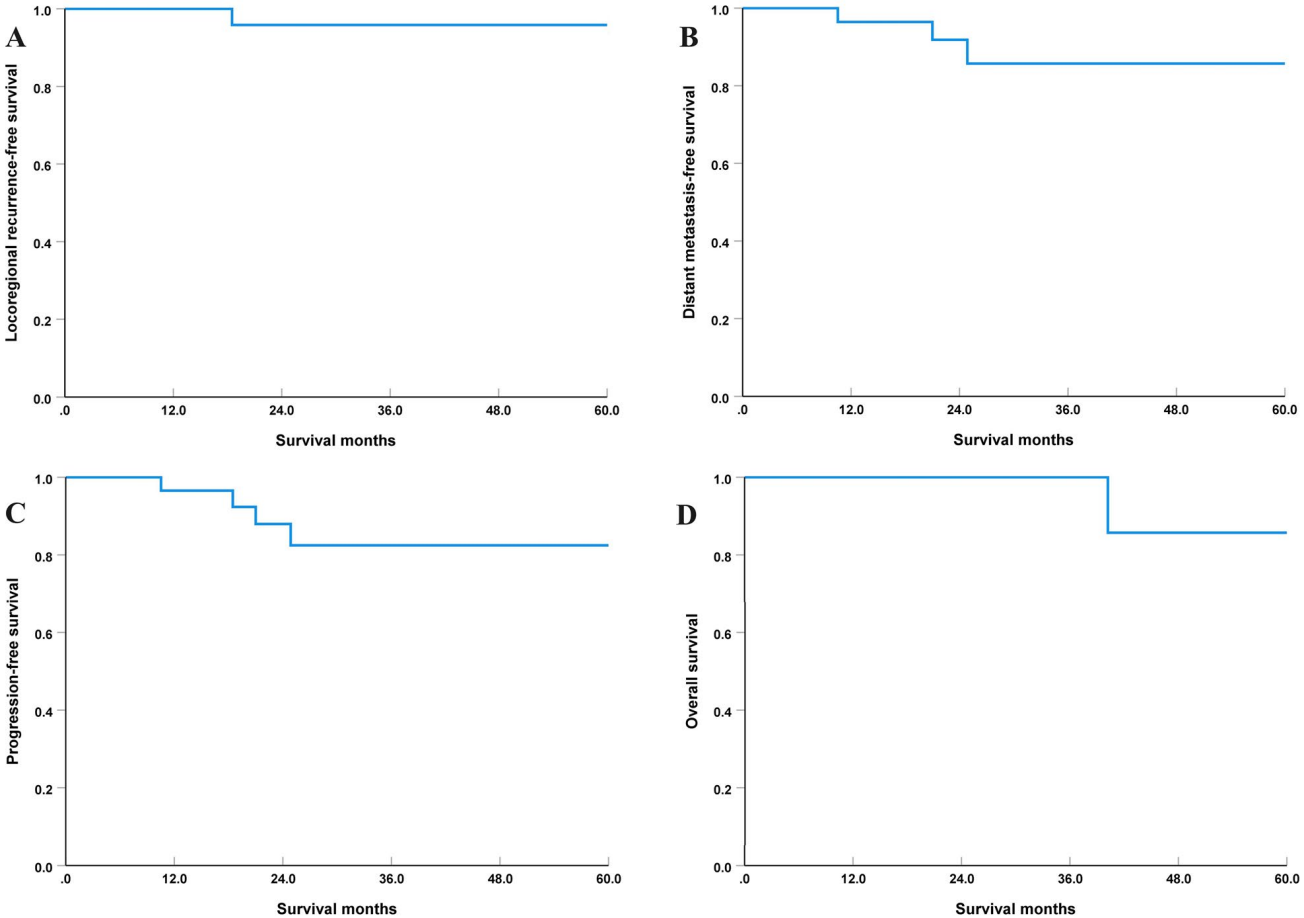
The locoregional relapse-free survival (A), distant metastasis-free survival (B), progression-free survival (C) and overall survival (D) in the cohort.

All patients underwent primary nasopharyngeal tumour and regional lymph node evaluation after three months of CCRT. There were eight patients who had CR (27.6%) and 21 patients who had PR (72.4%). The total response rate (CR + PR) was 100%. EBV-DNA was not detected in all patients. All patients had CR to the primary nasopharyngeal tumour and regional lymph nodes in the evaluation 6 months after CCRT. All patients were followed up every three months within three years of NPC diagnosis. The median follow-up was 32.1 months (range, 12.9–60.8 months). One patient experienced locoregional recurrence 18.5 months after the diagnosis of NPC. Three patients developed distant metastasis during follow-up, one patient had lung metastasis 21.0 months after the diagnosis of NPC, one patient had liver and retroperitoneal lymph node metastasis 24.8 months after the diagnosis of NPC, and one patient had liver and lung metastasis 10.5 months after the diagnosis of NPC. As of the current analysis, all four patients are still alive. A patient died of pulmonary infection 40.2 months after diagnosis of NPC, without any evidence of tumour recurrence or metastasis. The 3-year LRFS, DMFS, PFS and OS were 95.8%, 85.7%, 82.5% and 100%, respectively (Figure 1).

## Discussion

In this study, we investigated the efficacy and toxicity of lobaplatin-based CCRT in elderly patients with NPC. Our results showed that lobaplatin-based CCRT had satisfactory short-term survival outcomes and acceptable toxicities in this population.

With the acceleration of aging worldwide, the treatment of NPC in the elderly has become a major health burden, especially in the Chinese population. Due to the deterioration of physical functions, poor psychological endurance, as well as a higher number of comorbidities, and lacking sufficient social support in the elderly [4–6], the management of elderly NPC brings complex challenges to clinicians. Therefore, elderly patients are a heterogeneous group, and individual decisions are required in terms of treatment. Despite new advances in the management of NPC in recent years, elderly patients are often underrepresented in clinical trials [9–12]. In our study, 34.5% of patients developed grade 3 oral mucositis during CCRT. All patients had received the active nutritional intervention and the treatment compliance was generally high. The median weight loss of 4 kg during CCRT, was lower than that reported in the general NPC population (median weight loss of 4–7.85 kg) [27,28]. Therefore, for elderly NPC patients, careful monitoring and intensive support under the care of a multidisciplinary team should be instituted.

In several previous studies, 73.4–84.1% of patients were diagnosed with locally advanced-stage disease [21,29]. In our study, we also found 86.2% of the patients had stage III–IVA disease. The currently recommended CCRT regimen for patients with stage III–IVA NPC is cisplatin-based CCRT [16,17]. However, due to a lack of prospective studies, the treatment of the elderly still mainly refers to young patients. In the current clinical practice, approximately 60% of elderly NPC patients were treated with cisplatin-based CCRT [19,20,30]. However, most studies found no survival benefit of cisplatin-based CCRT but were associated with higher incidences of haematological and gastrointestinal toxicity compared to those treated with radiotherapy alone in the IMRT era [18,19]. The results from Mi et al. showed that the 3-year LRFS, DMFS, PFS and OS were 98.0%, 71.6%, 70.1% and 72.5% in those treated with cisplatin-based CCRT, respectively. However, the receipt of chemotherapy during IMRT had similar outcomes to those treated with IMRT alone but significantly increased gastrointestinal and haematological toxicity [18]. In addition, the study from Sommat et al. included 185 patients with elderly NPC (40% received cisplatin-based CCRT), the 5-year LRFS, PFS and OS were 80.6%, 51.4% and 64.5%, respectively [20]. In our study, the 3-year LRFS, DMFS, PFS and OS were 95.8%, 85.7%, 82.5% and 100% in those treated with lobaplatin-based CCRT, respectively. To our knowledge, this study was the first to investigate the role of third-generation platinum medication lobaplatin in elderly patients with NPC. The results of our study offer initial evidence that lobaplatin-based CCRT might be a promising alternative treatment modality in this population.

In terms of the distribution of causes of death, although elderly patients with NPC were more likely to die from treatment complications or other comorbidities than younger patients, it should be noted that 60–70% of patients still die from recurrence or distant metastatic diseases [21,29]. A recent study from Kou et al. included 583 elderly patients, they found that cisplatin-based CCRT was associated with better survival outcomes compared to those treated with IMRT alone in patients with a higher risk of disease recurrence [30]. Therefore, it is of great significance to explore high-efficiency and low-toxicity treatment strategies for elderly NPC patients.

Lobaplatin is a third-generation platinum antineoplastic agent. In recurrent and metastatic NPC, lobaplatin combined with docetaxel had clinical activity and acceptable treatment toxicity [31,32]. The disease control rate was 81.1–84.6% in this population. The main grade 3–4 treatment toxicity was haematological toxicity, including leukopenia (18–27%), anaemia (0–5.1%), thrombocytopenia (0–10.8%) and other toxicities were mild [31,32]. Several preclinical studies have shown that lobaplatin elicits treatment effects in NPC cells via inducing apoptosis, pyroptosis and cell cycle arrest [33–35]. In the CCRT setting, a phase 3 trial from Lv et al. showed that lobaplatin-based CCRT had non-inferior outcomes and fewer toxic effects compared to cisplatin-based CCRT [23]. However, we should notice that only patients aged <60 years were included in the above study. The 3-year LRFS, DMFS, DFS and OS were 90.7%, 88%, 80.3% and 92.1% in those younger patients treated with lobaplatin-based CCRT [23]. The present study included patients aged ≥65 years and found similar survival outcomes using lobaplatin-based CCRT in the elderly population compared to those with younger NPC. Our study also found that lobaplatin-based CCRT has extremely low gastrointestinal toxicity and nephrotoxicity. In addition, treatment compliance was also relatively high using the lobaplatin-based CCRT in our study. Therefore, lobaplatin might be a good candidate for CCRT in elderly NPC patients. However, we should also notice that the incidence of grade 3–4 thrombocytopenia (27.6% vs. 8%) and anaemia (10.3% vs. 2%) was higher in our study with elderly patients compared to those with younger patients [23]. Since the elderly have more potential comorbidities, lobaplatin-based CCRT for the elderly should mainly strengthen the monitoring of haematological indicators and improve individualized care.

Several limitations should be acknowledged in this study. First, 86.2% of patients receive IC, and the regimens have a certain heterogeneity due to physician biases. However, the prospective trials for elderly NPC patients may be hindered and our study reflects the therapeutic outcomes in real-world scenarios. Second, we only included a small sample size in this study. Therefore, the optimal treatment strategies for elderly NPC patients should be further investigated in the IMRT era. Third, we only reported the short-term survival in this population. The median follow-up was 32.1 months in this study and longer follow-up is required to confirm our results. Finally, 65.5% of patients in our study had ACCI ≥3. Due to the common issue of comorbidity in the elderly population, further studies are required to explore the significance of comorbidity in facilitating patient consultations and enhancing patient decision-making processes.

## Conclusions

In conclusion, our results demonstrate that lobaplatin-based CCRT is safe and feasible for elderly NPC patients, with satisfactory short-term survival outcomes and acceptable toxicities. Further investigation is required to determine the optimal treatment strategy for this population. A phase 2 trial is ongoing to investigate the role of lobaplatin-based CCRT on long-term survival and treatment toxicities in elderly NPC patients.

## Data Availability

Any request for data and material may be sent to the corresponding author.
